# Primary cardiac tumors in children: a center’s experience

**DOI:** 10.1186/s13019-016-0448-5

**Published:** 2016-04-11

**Authors:** Liyang Ying, Ru Lin, Zhan Gao, Jianchuan Qi, Zewei Zhang, Weizhong Gu

**Affiliations:** Department of Cardiac Surgery Children’s Hospital, Zhejiang University School of Medicine, Binsheng Load 3333#, Hangzhou, 310003 PR China; Department of Pathology Children’s Hospital, Zhejiang University School of Medicine, Binsheng Load 3333#, Hangzhou, 310003 PR China

**Keywords:** Cardiac tumor, Cardiac surgery, Pediatric

## Abstract

**Background:**

Cardiac tumors which may induce sudden death are rare entities with an autopsy frequency of 0.001–0.030 %. This study aims to analyze the characteristics and outcome of pediatric patients with primary cardiac tumors treated in our center.

**Methods:**

Sixteen patients with primary cardiac tumors treated at our center between January 2000 and December 2014 were included into this retrospective review. The patients’ age ranged from 1 day to 13 years (mean age, 46 months), with weight ranging from 3.2 to 45 kg (mean weight 17.5 kg). All patients were diagnosed by echocardiography, magnetic resonance imaging and computed tomography.

**Results:**

We did complete resection of the mass in 15 patients with cardiopulmonary bypass (CPB), whereas partial resection was done in one patient. Fifteen children recovered well, and one patient died of low cardiac output syndrome at 5 days after operation. Rhabdomyoma was the most frequent tumor type, followed by myxoma, fibroma, hemangioma; No malignant tumors were found.

**Conclusions:**

Echocardiography has provided consistent assessment of anatomy and function. Complete surgical resection is valuable treatment for cardiac mass when detected even in asymptomatic patients. Rhabdomyoma is the most frequent tumor type, followed by myxoma and fibroma.

## Background

Cardiac tumors which may induce sudden death [1, 2] are rare entities with an autopsy frequency of 0.001–0.030 % [3]. The majority of pediatric cardiac tumors are primary, originating in the heart itself, and benign. A review of the literature revealed that almost 40 % of the patients are detected incidentally through rountine prenatal screening with fetal ultrasonography [4]. The invention of cardiopulmonary bypass (CPB) in 1955 brought major changes and improvements to the management of heart tumors. Clinical findings of primary cardiac tumors vary in different ways. As surgical therapy is curative in most cases, early diagnosis is required to prevent complications; and different tumors location and cells type may induce a variety of therapy options and outcomes.

This study aimed to analyze the characteristics and outcome of pediatric patients with primary cardiac tumors treated in our center.

## Methods

Sixteen patients with primary cardiac tumors treated at our center between January 2000 and December 2014 were included into this retrospective review. All patients were diagnosed by echocardiography, magnetic resonance imaging (MRI) and computed tomography (CT) radiography.

We did complete resection of the mass in 15 patients with cardiopulmonary bypass (CPB), whereas partial resection was done in one patient. Two cases were done with right ventricle outflow tract enlargement by autologous pericardial after tumors excision. We repaired tricuspid valve reflux because of mass resection with one patient.

Patients were not discharged from intensive unit (ICU) until circulation and breathing were stable. Electrocardiogram, chest X-ray and echocardiography were done before patients left hospital. And patients were followed-up by phone interview and regular check after operation to assess late functional status.

## Results

The patients’ age ranged from 1 day to 13 years (mean age, 46 months), with weight ranging from 3.2 to 45 kg (mean weight 17.5 kg). The rate of the neonate accounted for 43.75 % (7/16) in this study. Patients’ characteristics, as well as tumors locations and histological type of tumors, are listed in Table 1. On admission, most of the patients were asymptomatic with cardiac murmur.

**Table 1:** Clinical data of primary cardiac tumors

Gender	Age(M)	Weight(Kg)	Symptom	Tumor location	Histological type
Female	50	15,5	Cardiac murmur	Tricuspid valve	Fibroma
Female	1	4.3	Cardiac murmur	Right ventricle	Hemangioma
Female	95	19	Asymptom	Left atrium	Myxoma
Male	100	40	Dyspnea	Left atrium	Myxoma
Female	1	4	Prepregnancy	Right ventricle	Fibroma
Female	157	45	Dyspnea	Left atrium	Myxoma
Female	2	5.5	Asymptom	Right ventricle	Rhabdomyoma
Male	1	5.5	Asymptom	Right ventricle	Rhabdomyoma
Male	1	3.5	Asymptom	Right ventricle	Rhabdomyoma
Female	6	8	Asymptom	Right ventricle	Rhabdomyoma
Male	1	3.7	Arrhythmia	Right atrium	Papillary fibroelastoma
Male	1	5.5	Asymptom	Right ventricle	Rhabdomyoma
Male	1	5.5	Cardiac murmur	Right ventricle	Fibroma
Female	156	57	Dyspnea	Right atrium	Myxoma
Female	156	50	Cardiac murmur	Right atrium	Cyst
Female	8	9.5	Asymptom	Right ventricle	Fibroma

All the patients were done under CPB, with the CPB time was 31 ~ 65 min (mean time 44 min) and aortic cross-clamping time was 21 ~ 53 min (mean time 34 min). Overall median ICU stay was 3 days (range, 1–10 days), with a median mechanical intermittent positive-pressure ventilation of 14 h (range, 3–65 h). 15 patients were successfully discharged from the hospital; one patient had supraventricular tachycardia which induced low cardiac output, and he died of low cardiac output syndrome at 5 days after right ventricle rhabdomyoma excision. Postoperative complications included low cardiac output in 2 patients, infections in 2, pleural effusion in 1 and atelectasis in 1. No thromboembolic and pericardial effusion occurred.

**Fig. 1:**
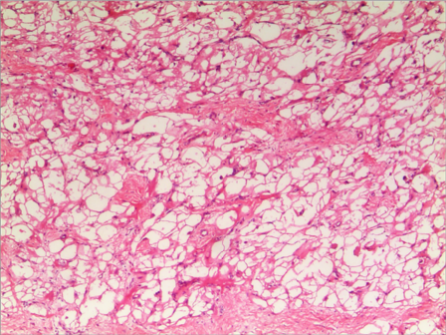
Rhabdomyoma contains cells that are filled with glycogen cytoplasm and extend to the periphery of the cell (haematoxylin and eosin, magnification*100)

**Fig. 2:**
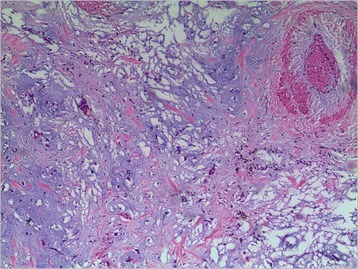
Myxoma cells arranged singularly and in cords, surrounded by abundant myxoid stroma and collagen-rich stroma (haematoxylin and eosin, magnification*100)

**Fig. 3:**
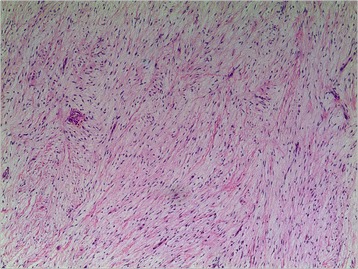
Fibroma:the spindle cells are arranged closely and can be seen to grow in the myocardium (haematoxylin and eosin, magnification*100)

Pathological analysis revealed that rhabdomyoma (Fig. 1) was the most frequent tumor histotype (6 cases, 37.5 %), followed by myxoma (4 cases, 25 %) (Fig. 2), fibroma (4 cases, 25 %), hemangioma (1 case, 6.25 %) (Fig. 3), Cyst (1 case, 6.25 %), and papillary fibroelastoma (1 case, 6.25 %). Right ventricle was the most mass location (9 cases, 56.25 %), followed by left atrium (3 cases, 18.75 %), right atrium (3 cases, 18.75 %) and tricuspid valve (1 case, 6.25 %).

At a mean follow-up of 5.44 years (range, 0.3–15 years), all the 15 patients are alive and recovered well, and majority of whom are the New York Heart Association class I. The ejection fraction (EF) was more than 50 % by echocardiography in all the 15 patients. There were no tumor recurrence and metastasis, and also no other complications such as valve reflux, right ventricular outflow tract obstruction.

## Discussion

Cardiac neoplasms are rare both in adults and children. The majority of tumors in adults are secondary, arising from noncardiac tissue, and either invading or metastasizing to the heart [5]. While the majority of tumors in the pediatric population are primary, arising from the heart itself.

Primary cardiac tumors may be asymptomatic with up to 12 % of cases found incidentally during evaluation of a related medical condition [6]. The clinical manifestation of the primary tumors may be related to tumor’s size and location, because as they grow, they can progressively obstruct cardiac chambers mimicking valve disease. So dyspnea may be followed by symptoms of heart failure. However, in the current literature on pediatric cardiac tumors, presence of symptoms does not affect overall survival, incidence of postoperative complications. In our study, 43.75 % of the patients were asymptom. Cardiac murmurs were found in four patients for the initial presentation when undergoing a routine heart check. Dyspnea occurred in three patients in whom tumors compressed the heart tightly. In this series, the neonate occupied 43.75 % of patients. Therefore, it is necessary to strengthen the prenatal fetal heart examination.

Echocardiography plays a very important role in the cardiac tumors diagnosis, which can evaluate the morphology, location and range of the tumor, also can assess the degree of blood flow obstruction caused by tumor. To achieve a complete diagnosis, in addition to echocardiography, magnetic resonance imaging (MRI) and computed tomography (CT) scan were necessary [7, 8]. Primary cardiac tumors should be considered as a possible diagnosis for all patients with embolization. Cardiac imaging has difficulty in categorizing intracardiac mass and some patients may have their mass classified as thrombus after pathological evaluation. A recent study [9] found a prevalence of cardiac thrombus in 13 of 84 patients with cardiac tumors. In our series, 6 patients had done MRI and CT and no one was diagnosed with embolization after operation. In the recent years, we had done MRI, even transesophageal echo, to confirm the diagnoses.

Though some cardiac tumors such as rhabdomyoma may potentially regress, surgery was considered as a prophylactic strategy to prevent mass-related potentially fatal complications which contained tumor embolization, severe valve regurgitation and ventricular arrhythmias. Furthermore, surgery for cardiac tumors in pediatric age carries an acceptable mortality risk [10–12]. Additionally, in children with benign cardiac tumors, there was no difference in terms of overall mortality and postoperative complications between complete and partial resection of the tumor other than myxoma, especially for those tumors that may spontaneously regress such as rhabdomyomas [13, 14]. The goal of surgery is to resect the entire mass with sufficient margins, which is almost always achievable with benign cardiac tumors. Rhabdomyomas are the notable exception to this rule. In patients who need surgery for a rhabdomyoma, subtotal excision to preserve surrounding structures may be reasonable, with the expectation of further stability or resolution [15]. By contrast, ventricular fibromas can usually be aggressively in which the fibromas is very extensive, heart transplantation might be needed [16]. In our study, one patient with rhabdomyoma located in the right ventricular outflow tract in whom partial resection was performed owing to big tumor. And we used fresh autologous pericardium to enlarge right outflow tract. At a follow-up of 5 years with this patient, the tumor regressed slowly.

Most of primary tumors resection in children requires CPB, especially when the mass is intracardiac. Surgical approach such as transatrial, transventricular and transaortic depends on the mass position. Surgical resection in the operation needs gentle manipulation of the heart to prevent mass fragmentation and embolization.

The pathological examination of excised cardiac masses in our series had showed the rhabdomyoma is the most frequent tumor in the pediatric age, followed by myxoma, fibroma, hemangioma, whereas the malignant tumors are very rare. Our results were consistent with literature [17–19] reported and obviously differ from other literatures [11, 12]. Morbidity of rhabdomyomas and fibromas was reported to be higher in the infancy, while myxomas are more frequent in older children [20]. The mortality in this series is 6.25 % (1/16) and no patient died during the follow-up; and no malignant tumors were found. No recurrence of a cardiac tumor occurred in these patients during the follow-up.

## Conclusion

The majority of primary cardiac tumors in children are benign and patients present in a variety of ways. Common presentations in this study included auscultated murmur and dispynea. Echocardiography has provided the consistent assessment of anatomy and function. CT and MRI are essential in distinguishing between cardiac tumors and embolization. Complete surgical resection is the most valuable treatment for cardiac mass when detected even in asymptomatic patients. In this surgical series, rhabdomyoma was the most frequent tumor, followed by myxoma, fibroma, and hemangioma, whereas no primary malignant cardiac tumors were found. No patients who died at a follow-up and no recurrence or reoperation occurred after the rumors resection.
